# Lymphoblastic lymphoma in two young siblings (coincidence or genetics?): two case reports

**DOI:** 10.1186/s13256-021-02977-1

**Published:** 2021-07-28

**Authors:** Naya Talal Hassan, Ebrahim Makhoul, Jafar Sallameh, Abdulmunem Ghanem, Samer Rajab, Waseem Ali, Zuheir Alshehabi

**Affiliations:** 1 Department of Dermatology, Tishreen University Hospital, Latakia, Syria; 2 Cancer Research Center, Tishreen University, Latakia, Syria; 3 Faculty of Medicine, Tishreen University, Latakia, Syria; 4 Department of Orthopedic Surgery, Tishreen University Hospital, Latakia, Syria; 5 Department of Pediatrics, Tishreen University Hospital, Latakia, Syria; 6 Department of Thoracic Surgery, Tishreen University Hospital, Latakia, Syria; 7 Department of Pathology, Tishreen University Hospital, Latakia, Syria

**Keywords:** Lymphoblastic lymphoma, Non-Hodgkin lymphoma, Familial cancer, Mediastinal mass, Case report

## Abstract

**Background:**

Non-Hodgkin lymphoma is the fourth most common malignancy in children, and it is not considered to be a hereditary disorder. However, it could affect members from the same family.

**Case presentation:**

We are presenting two cases of Caucasian female siblings who were diagnosed with mediastinal lymphoblastic lymphoma in the same year. The two young females were presented to the emergency department with respiratory symptoms. After doing radiological investigations and biopsies, they were diagnosed with lymphoblastic lymphoma. The elder sister died before confirming the diagnosis, and the other is on chemotherapy now, with good treatment outcomes.

**Conclusions:**

This case emphasizes the crucial role of precursor genetics in lymphoblastic lymphomas and suggests a strong relation between these genetics and age at symptom presentation. This is the first report of non-Hodgkin lymphoma in a pair of siblings in the pediatric population.

## Background

Among the homogeneous neoplasms, there are major changes that impact how these cases should be evaluated and diagnosed that have significant therapeutic effects as well as being of biologic interest [1]. Non-Hodgkin lymphoma (NHL) is the fourth most common malignancy in children [2]. It is not considered to be a hereditary disorder, but the occurrence of NHL lymphoma in the same family is called familial NHL [3]. Pediatric NHL shows important differences in the distribution of histologic subtypes to NHL observed in adults who have clinical characteristics represented by almost exclusively diffuse high-grade lymphomas and frequent extranodal involvement [2]. The prognosis for children with non-Hodgkin lymphoma has been improving over the last two decades [4].

Here we present a rare case of pediatric lymphoblastic lymphoma in two sisters, to emphasize the role of precursor genetics in lymphoblastic lymphomas and to suggest a strong relation between these genetics and age at symptom presentation. It is important that research on the genetic basis of pediatric lymphoblastic lymphoma continues, not only to establish its heritability but also to determine its possible impact on treatment and prognosis. We would like to report this case because of its rarity and its contribution to the literature.

## Case presentation

### Case 1

**Fig. 1:**
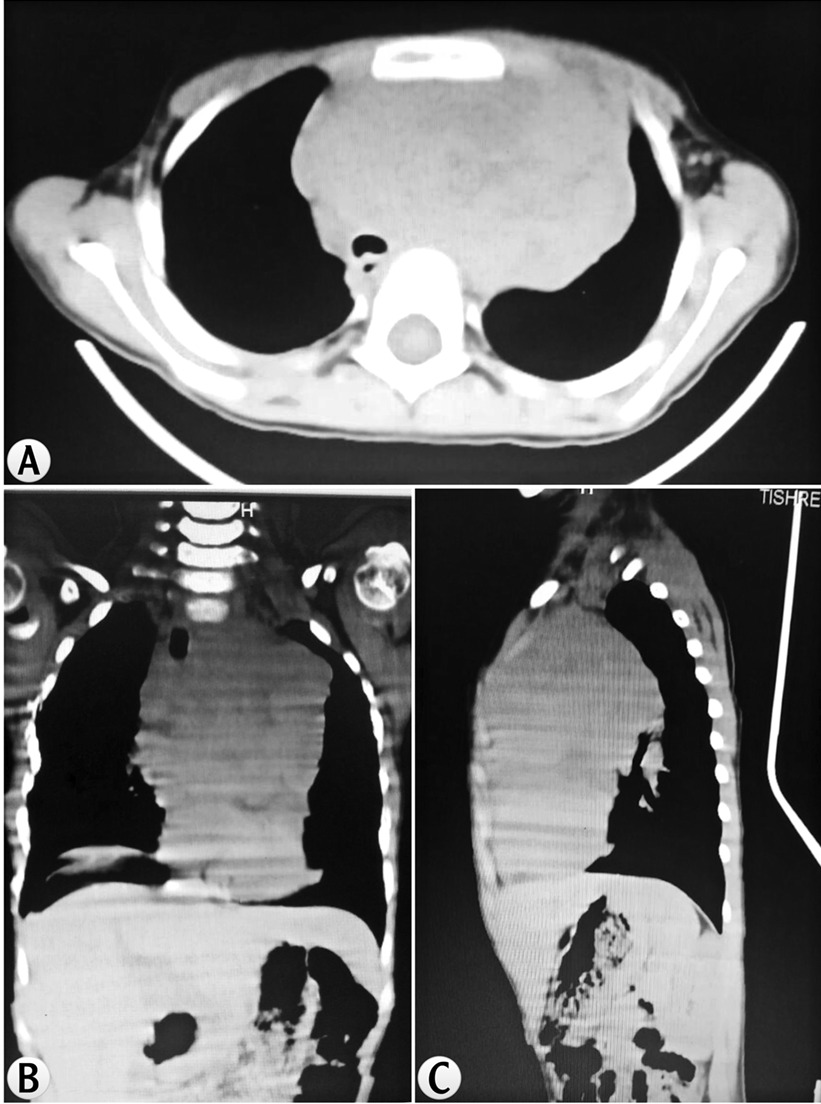
CT findings in case 1. The axial (**a**), coronal (**b**), and sagittal (**c**) CT planes show mediastinal expansion due to the big tumor mass, which is going up and out through the thoracic outlet, pushing the trachea to the right, narrowing the left bronchus

**Fig. 2:**
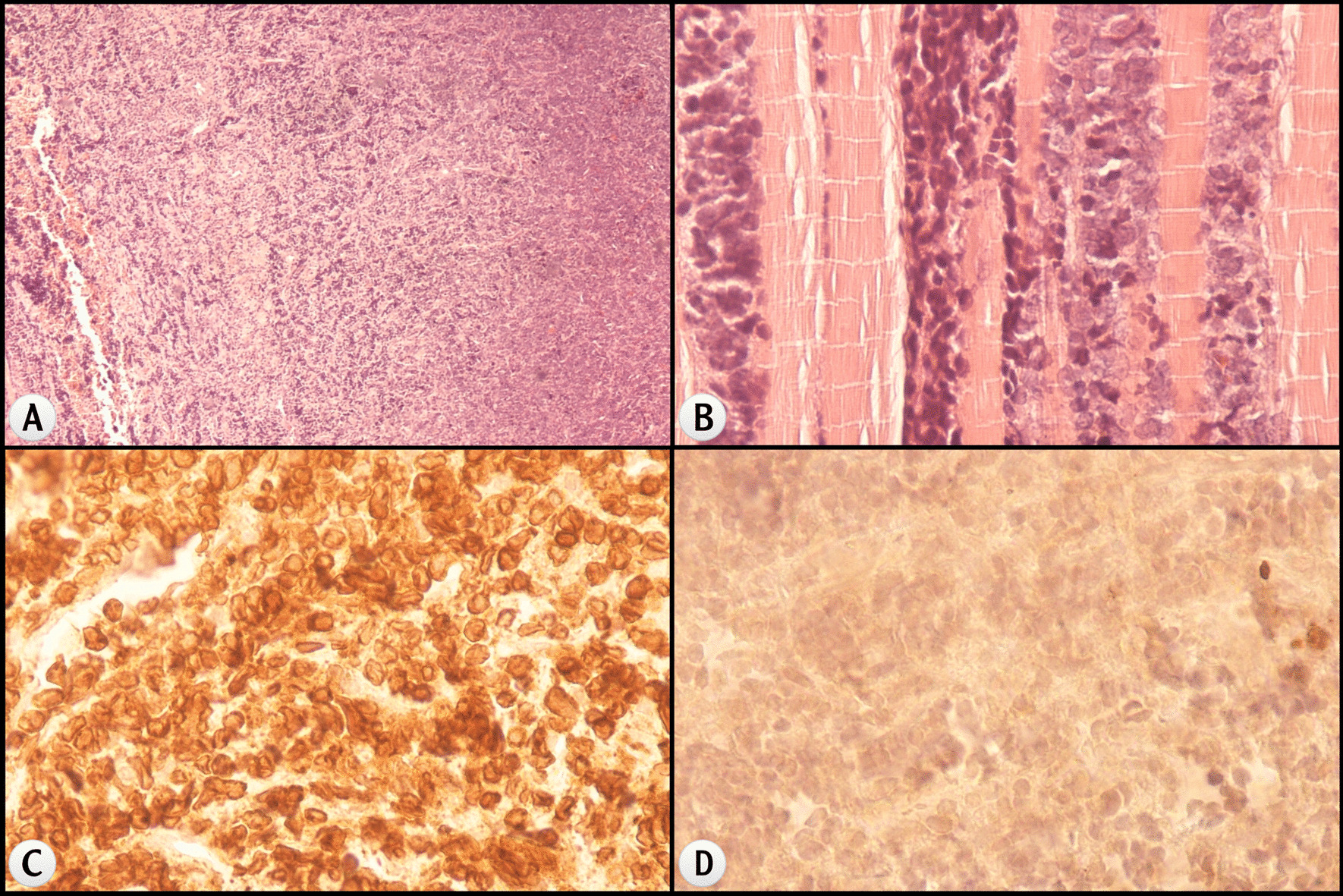
Histological findings of the mediastinal mass in case 1 (surgical biopsy). The histological features of the mediastinal mass revealed (**a**) [hematoxylin and eosin (H&E), ×40] T-cell lymphoblastic lymphoma. The malignant lymphocytes invade the strained muscle tissue (**b**) (H&E stain, ×40). Tumor cells show positivity for CD3 (**c**) and negativity for EMA (**d**)

A 5-year-old Caucasian female presented to our hospital with an intense dry cough and dysphagia for 2 weeks before the visit with no other symptoms. Her personal history was free of diseases. She was born second of her siblings by third-degree relative parents. Clinical examination revealed small enlarged lymph nodes at the level of the neck and immobile mass at the level of her right axilla with light edema. There were diffused café au lait spots all over her body. Auscultation revealed pulmonary wheeze sounds with a midsystolic murmur 2/6. The rest of the examination was within normal limits. Her weight (W) was 20  kg and her height (H) 110  cm. Complete cellular blood count (CBC) was completely normal. Because chest X-ray (CXR) showed a mass in the mediastinum, chest computed tomography (CT) was performed. Chest CT revealed a big mass along the mediastinum (Fig. 1). She underwent a surgical biopsy. The histological results (Fig. 2) revealed T-cell lymphoblastic lymphoma, and the malignant lymphocytes showed positivity for CD3 and terminal deoxynucleotidyl transferase (TdT), whereas CD20, myogenin, cytokeratins (CK), epithelial membrane antigen (EMA), desmin, and WT1 were negative. Ki67 was 50%. The girl went into a coma during the resection surgery and stayed in the intensive care unit (ICU) for 10 days, then died from cardiac arrest. We suggest that the fatality was due to compression on the air track by the mass after the anesthesia.

### Case 2

**Fig. 3:**
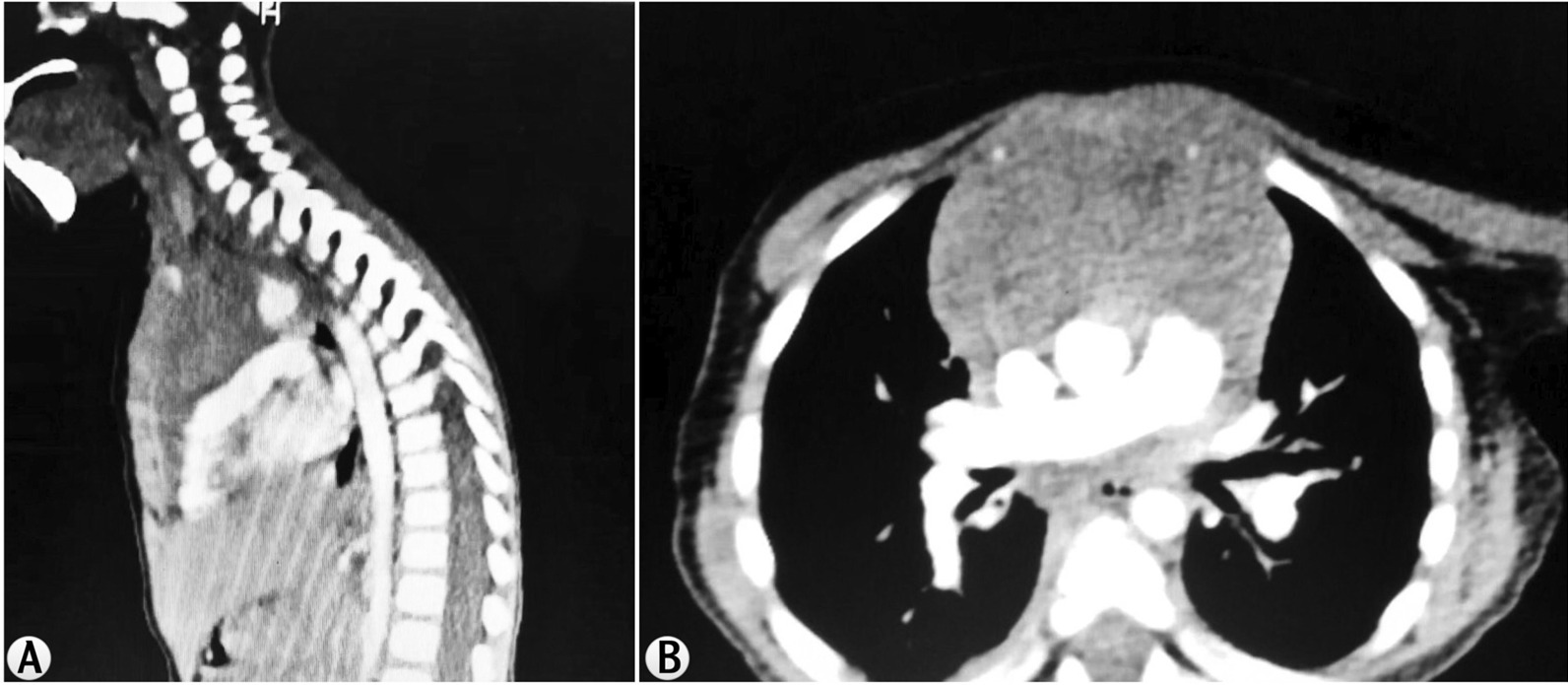
CT findings in case 2. The axial (**a**) and sagittal (**b**) CT planes show a large mediastinal mass rounding the neck vessels measuring 53 × 38 mm, extending to the thoracic cave around the branching of bronchi measuring 30 × 29 mm compressing both bronchi

**Fig. 4:**
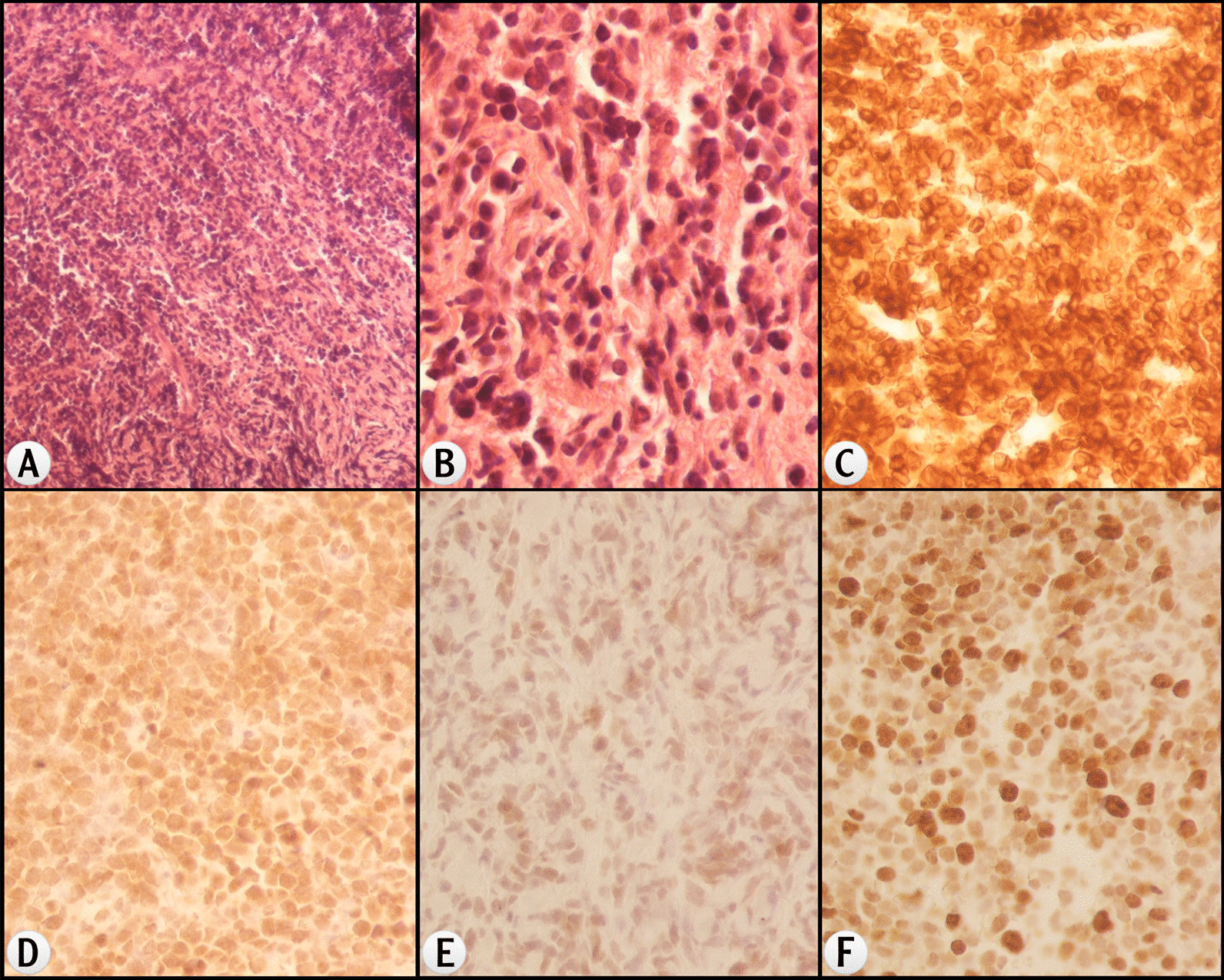
Histological findings of the mediastinal mass in case 2 (fine-needle biopsy). **a** (H&E, ×100) and **b** (H&E, ×600) show the malignant lymphocytes, which are CD3 (**c**) and TdT (**d**) positive, WT1 (**e**) negative. Ki67 is 50% (**f**)

An 18-month-old Caucasian female was admitted to a private clinic with a productive cough that was diagnosed mistakenly as bronchitis. Based on that diagnosis, she took many medications (antibiotics and pain relievers) and showed partial improvement. She came to our hospital because of the increasing dyspnea. Her personal history was free of diseases. She was born third of her siblings by third-degree relative parents. Clinical examination revealed the following: diffused café au lait spots all over her body and congenital Mongolian spots with enlarged submaxillary lymph nodes without any palpable visceral enlargement. Chest auscultation revealed diffuse pulmonary crackles. The rest of the examination, laboratory tests, brain CT, and bone marrow biopsy were all within normal limits. Her weight (W) was 10 kg and her height (H) 78 cm with head circumference 48 cm. Complete cellular blood count (CBC) was completely normal. Computed tomography (CT) revealed an enlarged palatine tonsil and enlarging in the mastoid lymph nodes and submaxillary lymph nodes. The nodes measured no more 11 mm. CT also showed a mass in the mediastinum (Fig. 3). In addition, there was a hyperplasia in the thymic area extending to the front of the heart. Mild hepatomegaly and enlarged paraaortic lymph nodes were also found. The histological results (Fig. 4) revealed T-cell lymphoblastic lymphoma with positivity for CD3 and TDT, whereas CD20, myogenin, CK, EMA, Desmin, and WT1 were negative. Ki67 was 50%. The girl has been undergoing chemotherapy (cytarabine, vincristine, ifosfamide) till now, and she is getting well, with no respiratory symptoms.

## Discussion and conclusions

It is estimated that 2% of (NHL) cases are familial. This risk of lymphoma occurring in the same family members varies depending on multiple factors, including histopathological type, age at diagnosis, sex, and familial relationship [3]. It is found that male relatives are affected more than females [5]. The incidence of NHL in siblings has been noticed for a long time; it is found that the lifetime cumulative risk of NHL in siblings of a patient with NHL was 1.6%. The lifetime risk was higher when NHL was diagnosed in a sister than in a brother [3]. Most of the common histopathological types of NHL showed familial incidence. Very high familial risk of histological subtypes were found for lymphoplasmacytic, mantle cell, and cutaneous T-cell lymphomas [3]. In the literature review, we found multiple cases of siblings with NHL (some are as case reports and others included in cross-sectional and other research studies). In 2006, Loves *et al.* reported a family in which three male siblings developed NHL [6]. Those three siblings had different pathological types of lymphoma and were diagnosed at ages 45, 56, and 52 years. Primary gastric NHL, which occurred in two sisters and their father, was also reported by Hayoz *et al*. in 1993 [7]. The ages of two sisters at diagnosis were 53 and 46 years, and their father’s age was 78 years. Their family had no history of immunodeficiency disorder or cancer. To our knowledge, our case is considered to be the first report of lymphoblastic lymphoma in a pair of siblings in the pediatric population. Some studies found that the diagnosing interval for NHL/NHL sibling pairs is about 1–4 years [8]. In our case, the interval between the appearance of symptoms in the two sisters was very short (less than 1 year). It is estimated that mediastinal non-Hodgkin lymphomas represent about 5% of all non-Hodgkin lymphomas. Mediastinal lymphomas can cause symptoms such as fever, malaise, chest pain, or other symptoms related to compression of adjacent mediastinal structures; however, they might be asymptomatic [9]. Besides the site of tumor, the symptoms and the immune stains were totally similar in the two cases. The occurrence of the disease in the same family leads to many questions about sharable factors that family members could have. These factors may include genetic factors such as tumor formation in Li–Fraumeni syndrome, or immune deficiency disorders, which are very rare. Other important factors are environmental factors such as viral infections like EBV, HIV, or CMV or chemical poisoning and pollution. The two girls have one older sister who is healthy. In our case, the parents are third-degree relatives, and both of the two girls have more than six café au lait spots. This type of spots usually results from recessive hereditary syndromes such as constitutional mismatch repair deficiency syndrome (CMMRD). Although there was no family history of genetic disorders and the girls were previously healthy before NHL diagnosis, the occurrence of the disease at the same time at the same site at this very young age and the presence of the same skin disorders in the two patients may indicate genetic disorders. CMMRD is a syndrome characterized by formation of different types of childhood tumors (hematological, neurological, colorectal, etc.) as well as cutaneous features of neurofibromatosis 1 (NF-1) such as café au lait spots [10]. About one-third of CMMRD patients develop hematological malignancies, and T-cell non-Hodgkin lymphomas, mainly of mediastinal origin, are the most common hematological malignancy [11]. The two patients in our case did not undergo genetic testing because these tests are not available now in our hospital. We suggest that the familial occurrence of the disease might trigger lymphoma formation at a younger age and at unusual sites. However, the prognosis of the disease appears to be the same in familial and nonfamilial cases. The genetic factors are one of the most important factors that affect NHL formation. Although we do not suggest performing scanning of a patient’s siblings when diagnosing NHL, we believe that more research should be done to determine the exact mechanism of how genetics are responsible for lymphoma formation in siblings and how this would affect the diagnosis and treatment of the disease as well as the genetic counseling for affected families.

## Data Availability

Data mentioned in this case report are available to the reviewers if required.

## Abbreviations

NHL: Non-Hodgkin lymphoma
CBC: Complete blood count
CXR: Chest X-ray
CT: Computed tomography
TdT: Terminal deoxynucleotidyl transferase
CK: Cytokeratins
EMA: Epithelial membrane antigen
ICU: Intensive care unit
EBV: Epstein–Barr virus
HIV: Human immunodeficiency virus
CMV: Cytomegalovirus
CMMRD: Constitutional mismatch repair deficiency syndrome
